# Prenatal Coffin-Siris Syndrome: Expanding the Phenotypic and Genotypic Spectrum of the Disease

**DOI:** 10.1177/10935266231210155

**Published:** 2023-11-19

**Authors:** Sini Keskinen, Teija Paakkola, Mirjami Mattila, Marja Hietala, Hannele Koillinen, Jukka Laine, Maria K. Haanpää

**Affiliations:** 1 Tyks Laboratories, Genomics, Clinical Genetics, Turku University Hospital, Turku, Finland; 2 Northern Finland Laboratory Centre NordLab and Oulu University Hospital, Oulu, Finland; 3 Department of Obstetrics and Gynecology, Turku University Hospital, Turku, Finland; 4 Department of Clinical Genetics, Turku University Hospital, Turku, Finland; 5 Institute of Biomedicine, University of Turku, Turku, Finland; 6 Department of Pathology, Turku University Hospital, Turku, Finland

**Keywords:** Coffin-Siris syndrome, CSS, prenatal, *SMARCB1*, *ARID1A*

## Abstract

Coffin-Siris syndrome is an autosomal dominant disorder with neurological, cardiovascular, and gastrointestinal symptoms. Patients with Coffin-Siris syndrome typically have variable degree of developmental delay or intellectual disability, muscular hypotonia, dysmorphic facial features, sparse scalp hair, but otherwise hirsutism and fifth digit nail or distal phalanx hypoplasia or aplasia. Coffin-Siris syndrome is caused by pathogenic variants in 12 different genes including *SMARCB1* and *ARID1A*. Pathogenic *SMARCB1* gene variants cause Coffin-Siris syndrome 3 whereas pathogenic *ARID1A* gene variants cause Coffin-Siris syndrome 2. Here, we present two prenatal Coffin-Siris syndrome cases with autosomal dominant pathogenic variants: *SMARCB1* gene c.1066_1067del, p.(Leu356AspfsTer4) variant, and a novel *ARID1A* gene c.1920+3_1920+6del variant. The prenatal phenotype in Coffin-Siris syndrome has been rarely described. This article widens the phenotypic spectrum of prenatal Coffin-Siris syndrome with severely hypoplastic right ventricle with VSD and truncus arteriosus type III, persisting left superior and inferior caval vein, bilateral olfactory nerve aplasia, and hypoplastic thymus. A detailed clinical description of the patients with ultrasound, MRI, and *post mortem* pictures of the affected fetuses showing the wide phenotypic spectrum of the disease is presented.

## Introduction

Coffin-Siris syndrome (CSS, OMIM 135900; “the fifth digit syndrome”) is a well characterized multiple congenital anomaly/intellectual disability syndrome. Classical features are fifth digit/nail hypoplasia, coarse facial features, and different organ-system related anomalies. CSS was first described in 1970^1^ and in 2004 three unrelated Finnish patients were reported.^2^ Approximately 7% to 12% of CSS patients have pathogenic *SMARCB1* variants and 7% have pathogenic *ARID1A* variants.^3,4^ CSS classifies further into twelve subgroups defined by the genetic etiology. Pathogenic *SMARCB1* gene variants cause CSS3 (OMIM 614608) and pathogenic *ARID1A* gene variants cause CSS2 (OMIM 614607).

*SMARCB1* gene encodes a core subunit of Brahma-related gene 1 (BRG1)—and Brahma (BRM)—associated factor complex (BAF complex) that functions in ATP-dependent chromatin remodeling^5^ and thus affects the regulation of gene expression and differentiation.^6^ *ARID1A* gene encodes a component of neural progenitors-specific chromatin and neuron-specific chromatin BAF remodeling complexes that function during neural development.^7^ A switch from progenitor to a postmitotic chromatin remodeling mechanism takes place as neurons exit the cell cycle and commit to their mature adult state.^7^ CSS encompasses a wide range of phenotypes and abilities caused by pathogenic variants in the BAF complex, which is often referred to as BAFopathy. CSS patients have typical craniofacial and skeletal features, structural brain abnormalities, muscular hypotonia, seizures, and can have genitourinary, gastointestinal and cardiovascular complications.^3,8^

## Case Report

### Clinical Description of Fetus 1

**Figure 1.**
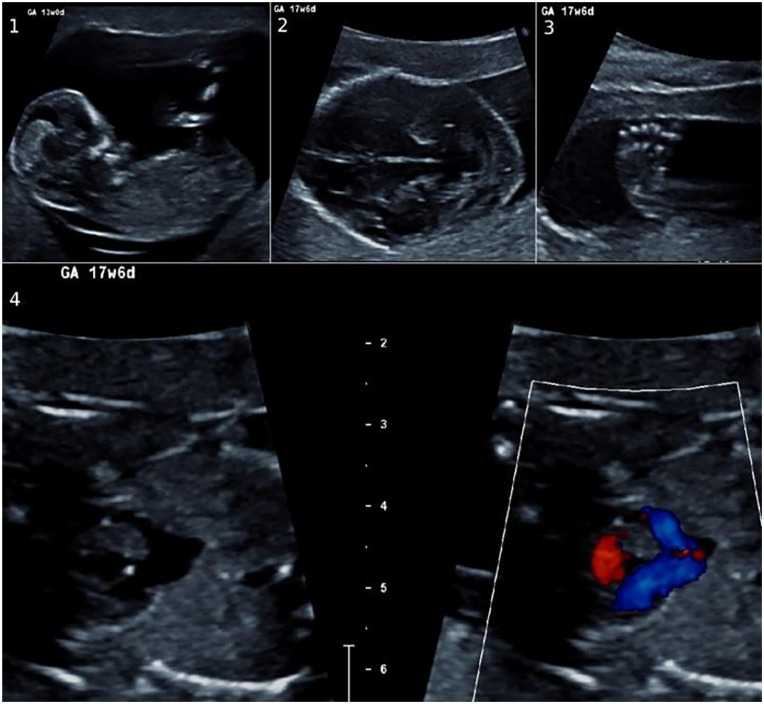
Fetus 1: ultrasound images. Fetus 1 with (1) elevated nuchal fold thickness, (2) absent cavum septum pellicidum, (3) club foot, and (4) functionally univentricular heart.

A 38-year-old woman (G1PO) was referred to the Department of Obstetrics. The first trimester screening was abnormal at 12 weeks of gestation. The nuchal fold thickness was elevated 5.5 mm (Figure 1), and the risk for trisomy was 1:6. A villus biopsy was taken at 13 weeks of gestation and the PCR test for trisomies 21, 13, and 18, and the chromosomal microarray analysis results were reported normal in a male fetus. At week 17 of gestation in an ultrasound there were multiple abnormalities; a functionally univentricular heart, bilateral club-foot, and the cavum septum pellicidum could not be visualized normally (Figure 1, Supplemental Table 1). The pregnancy was terminated.

**Figure 2.**
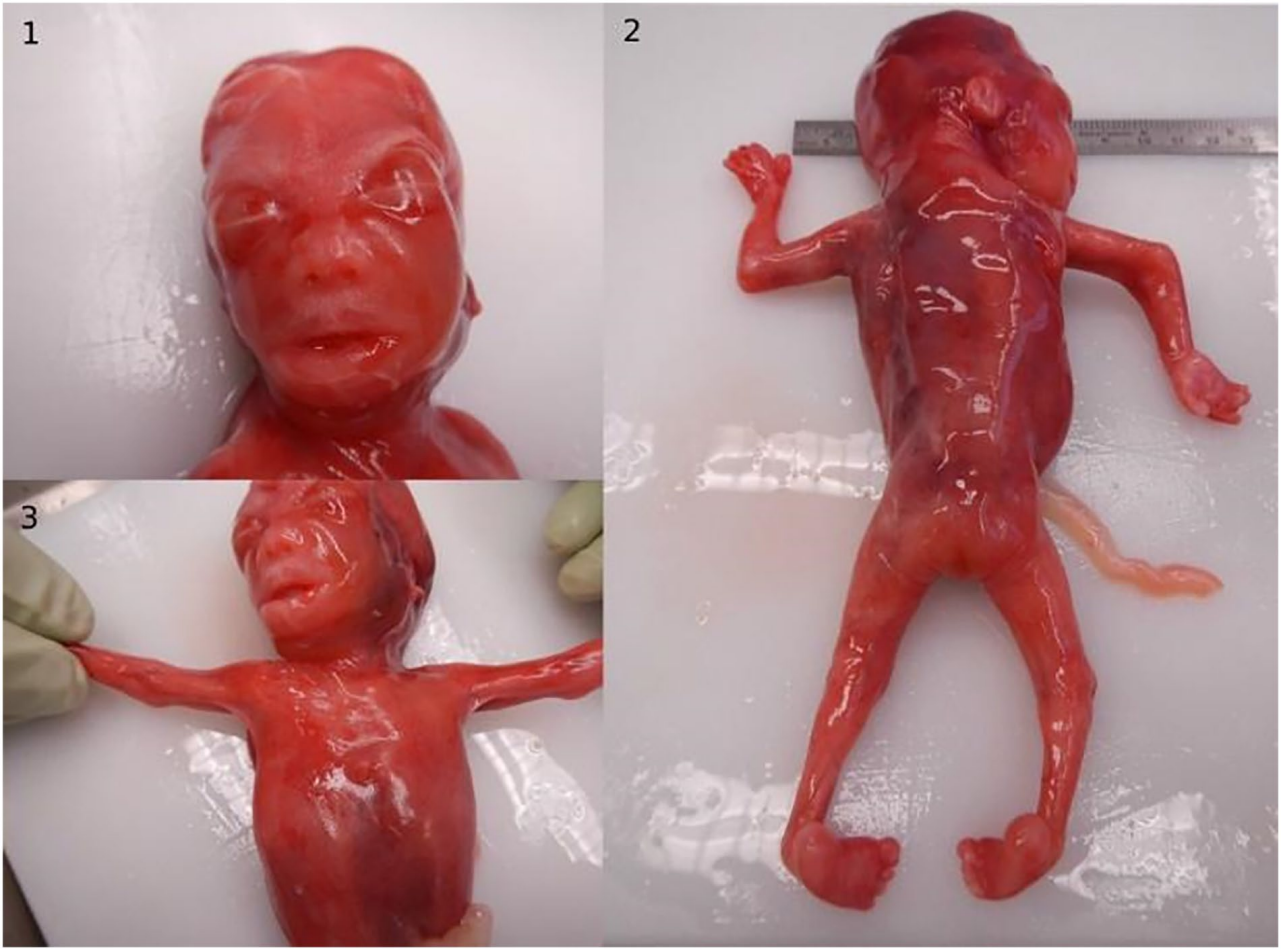
Fetus 1: autopsy photographs. Fetus 1 with (1) facial dysmorphic features, (2) club feet, and (3) upper limb pterygia.

In autopsy the fetus presented with facial dysmorphism, various congenital malformations including narrow waste, upper limb pterygia, slightly short lower limbs with club-feet, corpus callosum agenesis, frontal lobe hypoplasia, cleft palate, hypoplastic thymus, and severely hypoplastic right ventricle with VSD and truncus arteriosus (type III) and persisting left superior and inferior caval vein (Figure 2, Supplemental Table 1).

### Clinical Description of Fetus 2

**Figure 3.**
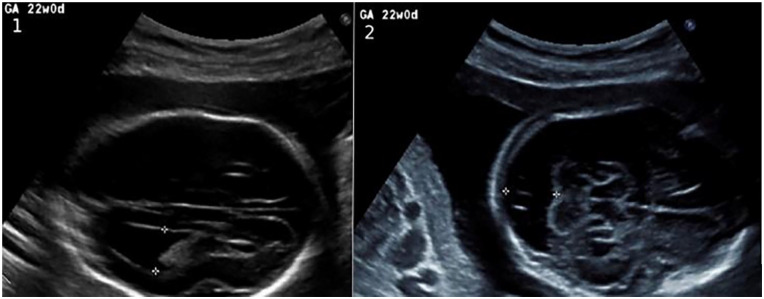
Fetus 2: ultrasound images. Fetus 2 with (1) dilated lateral ventricles of 12 mm and (2) mega cisterna magna of 18.5 mm.

A 32-year-old woman, who had 1 previous healthy child and 2 previous early miscarriages (G5P1) was referred to the Department of Obstetrics. At 20 + 6 weeks of gestation of a male fetus, an ultrasound scan revealed enlarged posterior fossa, wide lateral ventricles, and the cavum septum pellicidum could not be visualized normally (Figure 3; Sup-plemental Table 1). The PCR test for trisomies 21, 13, and 18 and the chromosomal microarray analysis results were reported normal. The pregnancy was terminated. The MRI showed agenesis of the corpus callosum and a suspicion of either cerebellar hypoplasia or a mega cisterna magna (Figures S1-S3).

**Figure 4.**
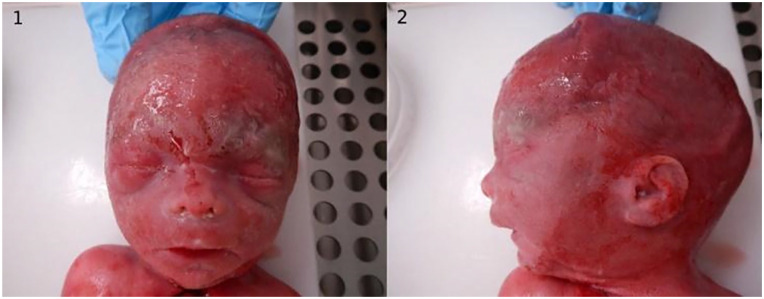
Fetus 2: autopsy photographs. Fetus 2 with (1) and (2) mild facial dysmorphic features.

In autopsy the fetus presented with mild dysmorphic features and central nervous system (CNS) malformations including corpus callosum agenesis, Dandy-Walker malformation, and hydrocephalus (Figure 4; Supplemental Table 1).

Detailed description of the autopsy findings is presented in the Supplemental Data.

## Results

Fetus 1 presented with a heterozygous de novo deletion c.1066_1067del, p.(Leu356AspfsTer4) in *SMARCB1* gene that caused a frameshift. The conserved heterozygous variant was classified as pathogenic.

Fetus 2 presented with a de novo heterozygous deletion of 4 nucleotides at splicing site of intron 4 and exon 5 in *ARID1A* gene. The heterozygous variant c.1920+3_1920+6del was classified as likely pathogenic.

Detailed description of the variant classification and methods are presented in Supplemental Data.

## Discussion

The prenatal CSS diagnosis is rare and there is need for better phenotypic description of the prenatal cases that help to guide the obstetrician and the clinical geneticist to manage the patient and give the appropriate genetic counseling. In previously published prenatal cases intrauterine growth restriction was detected in an ultrasound for 50% of fetuses with *SMARCB1* pathogenic variants and for 13% of fetuses with *ARID1A* pathogenic variants.^9-11^ In our study the ultrasound measurements of the fetuses were in keeping with gestational weeks (Supplemental Table 1). The first fetus had multiple malformations and the second fetus had only CNS malformations and mild dysmorphic features. The postnatal CSS related to *SMARCB1* have been described to be more severe in developmental delay and complications.^3,4,8^ In our study fetus 1 (*SMARCB1*) had a more severe phenotype compared with fetus 2 (*ARID1A*). A recently reported fetal cohort with *SMARCB1* pathogenic variants did not show more severe cardiac manifestations compared to other pathogenic variants and the fetuses with *ARID1A* pathogenic variants were more likely to perish in the neonatal period than the non-*ARID1A* fetuses.^11^

Both fetuses had typical dysmorphic features including hypertelorism and micrognathia as well as corpus callosum agenesis. The CNS malformations have been reported frequently in CSS.^3,4,8,12^ The corpus callosum agenesis, hydrocephalus, and dandy walker malformation have been described previously.^9-13^ A recently published study reported similar symptoms to fetus 1 with olfactory bulb hypoplasia and absent thymus.^9^ These novel prenatal findings in our study combined with the previous study suggest that these abnormalities in the olfactory nerves and thymus can be part of the phenotypic spectrum of CSS.

Fetus 1 had a severely hypoplastic right ventricle with VSD and truncus arteriosus (type III) and persisting left superior and inferior caval vein. In a larger fetal cohort of 44 fetuses with Coffin-Siris associated genes, 80% of fetuses with *ARID1A* pathogenic variant had a cardiovascular anomaly, and 33% of fetuses with *SMARCB1* pathogenic variant had cardiovascular anomalies.^11^ Only 1 fetus with a *ARID1A* pathogenic variant had a hypoplastic right heart and 2 had a hypoplastic left heart syndrome.^11^ The research group also reported hypoplastic left heart syndrome in 1 fetus with ARID1B and 2 fetuses with SMARCA4 pathogenic variants.^11^ Also a case with prenatal CSS related to SMARCC2 with tetralogy of Fallot has been reported.^14^

The analysis of clinically significant genes of fetus 1 showed a frameshift causing variant in *SMARCB1* as the cause for CSS3 and a small deletion in *ARID1A* as the cause of CSS2 in fetus 2. Patients with CSS have been reported with various types of *SMARCB1* gene variants such as stop-gain, frameshift insertion/deletion, splice site changes, and copy number variations.^3,15^ Interestingly, *SMARCB1* gene duplications and deletions which cause a frameshift in the reading frame of the gene transcript are not necessarily degraded by nonsense-mediated mRNA decay but instead lead to malfunctioning protein products.^15^ Pathogenic gene variants described in CSS have been dominant negative or gain-of-function variants.^5,16,17^ These variants often locate at the end of the gene transcript in exons 8 and 9 in Sucrose/Non-Fermenting domain 5 (SNF5 domain) like our finding in *SMARCB1*. The *SMARCB1* variant locates in the core component of BAF complex in SNF5 domain similarly to previously reported pathogenic *SMARCB1* gene variants.^3,16,17^ The novel *ARID1A* deletion c.1920+3_1920+6del on the other hand presumably leads to a truncated protein product due to the interference at the splicing site during gene transcription thus negatively affecting the function of the BAF complex. Mammalian BAF complex contains up to 15 subunits which are encoded by gene families and can be replaced by their paralogues leading to hundreds potential assemblies.^6^ Many of the resulting complexes are unique to specific tissues or biological functions such as neural development and function, heart development, muscle development, and embryonic stem cell pluripotency.^6^ The function of BAF complex is not currently known, however, it is hypothesized that genes in the BAF complex associate with transcription factors that function in neurodevelopment.^4^ Due to the complexity of the BAF complex, the clinical manifestations are variable, a factor that is common to CSS.

Our valuable fetal analyses of CSS with pathogenic *SMARCB1* and *ARID1A* gene variants bring new insights to the matter further affirming the importance of intact SNF5 domain of *SMARCB1* gene^3^ as well as the importance of *ARID1A* as BAF-specific stabilizing subunit^18^ and their role especially in brain abnormalities.

The CNS malformations and serious cardiac malformations are recurrent malformations in prenatal CSS cases. Serious cardiovascular and CNS malformations are observed in prenatal ultrasound whereas the typical craniofacial features and skeletal anomalies are more challenging to distinguish. Dysmorphic features are seen in the autopsies of fetuses. The exact diagnosis of CSS during pregnancy is rare but the genetic testing for fetuses has increased significantly. Thus, a detailed molecular, radiological, and pathological examination remains crucial especially with prenatal cases.

## Supplemental Material

sj-docx-1-pdp-10.1177_10935266231210155 – Supplemental material for Prenatal Coffin-Siris Syndrome: Expanding the Phenotypic and Genotypic Spectrum of the Disease Supplemental material, sj-docx-1-pdp-10.1177_10935266231210155 for Prenatal Coffin-Siris Syndrome: Expanding the Phenotypic and Genotypic Spectrum of the Disease by Sini Keskinen, Teija Paakkola, Mirjami Mattila, Marja Hietala, Hannele Koillinen, Jukka Laine and Maria K. Haanpää in Pediatric and Developmental Pathology
