# Supplementary material for: Prenatal Coffin-Siris Syndrome: Expanding the Phenotypic and Genotypic Spectrum of the Disease
Source: Pediatr Dev Pathol. 2023 Nov 19;27(2):181–6. doi: 10.1177/10935266231210155 (PMC11015708; doi:10.1177/10935266231210155)
Supplement: sj-docx-1-pdp-10.1177_10935266231210155 – Supplemental material for Prenatal Coffin-Siris Syndrome: Expanding the Phenotypic and Genotypic Spectrum of the Disease [file sj-docx-1-pdp-10.1177_10935266231210155.docx]

Supplementary data

Autopsy of the fetus 1:

In autopsy the measurements were in keeping with the gestational weeks (table S1). The fetus had a cleft palate, upper limb pterygia, narrow waste and slightly short lower limbs with club-feet (Fig. 2). The fetus had dysmorphic facial features including hypertelorism, swollen orbitas, broad and depressed nasal bridge, full lips, wide mouth, micrognathia and low-set posteriorly rotated ears. The fetus had frontal lobe hypoplasia, agenesis of the corpus callosum and bilateral olfactory aplasia. The fetus had hypoplasia of the thymus. The lungs lobulation was normal. In the heart, the auricle of the right atrium was enlarged and the left auricle was rudimental. The tricuspidal valve was rudimental and the right ventricle was extremely hypoplastic. The right and left ventricles formed one large chamber as a large ventricular septal defect covered virtually the whole septum. Another set of inferior and superior vena cava were located on the left side of the heart. There was a truncus arteriosus located on the left ventricle leading to the pulmonary arteries. Truncus arteriosus was of type three of the Collet and Edwards system, where right and left pulmonary arteries arise independently from either side of the truncus. There was no ductus arteriosus present.

Autopsy of the fetus 2:

In autopsy the measurements (weight, crown-rump length, and toe-heel length) of the fetus were in keeping with 24-25 weeks (vs. observed h23+0) (table S1). The fetus had mild dysmorphic features including micrognathia, hypertelorism, broad nasal tip, anteverted nares and broad philtrum (Fig. 4). The fetus had corpus callosum agenesis, hydrocephalus and a Dandy-Walker malformation. The fetus had otherwise normal structures.


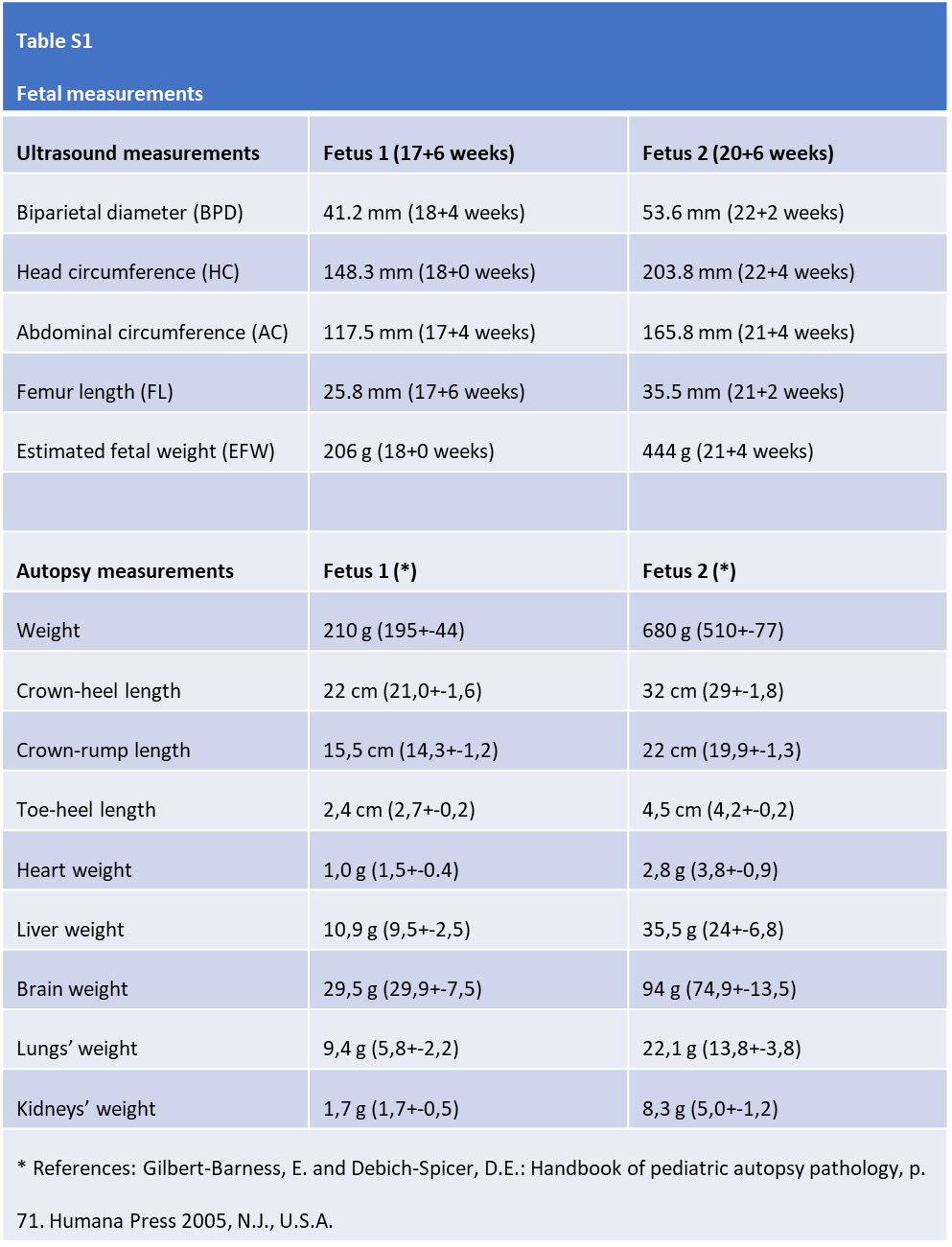


Figure S1-S3: Fetus 2: Magnetic resonance imaging showing interhemispheric separation, corpus callosum agenesia and hypoplastic cerebellum with megacisterna magna.


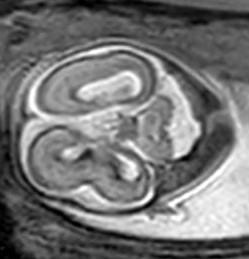


Figure S1


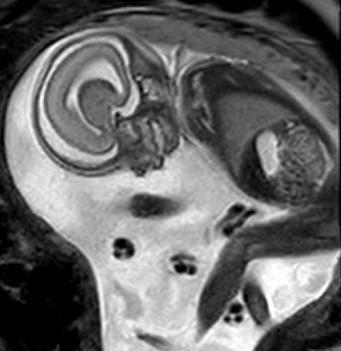


Figure S2


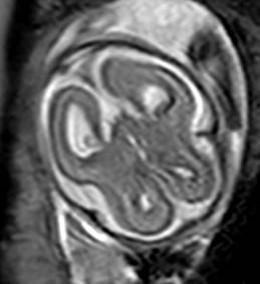


Figure S3

Results:

Fetus 1 presented with a heterozygous *de novo* deletion c.1066_1067del, p.(Leu356AspfsTer4) in *SMARCB1* gene that caused a frameshift. The conserved heterozygous variant was classified as pathogenic (ACMG class 5: PVS1, PS2, PM2). The discovered rare *SMARCB1*-variant was absent in the Genome Aggregation Database (GnomAD) but was reported once in Clinical Variant Database as a likely pathogenic variant without phenotypic description (ClinVar: VCV000532963.5).

Fetus 2 presented with a *de novo* heterozygous deletion of four nucleotides at splicing site of intron 4 and exon 5 in *ARID1A* gene. Deletion c.1920+3_1920+6del at the splicing site causes four nucleotides to be removed from intron 4 and affects negatively to the splicing of the transcript most likely leading to a truncated protein. The heterozygous *ARID1A* variant was classified as likely pathogenic (ACMG class 4: PS2, PM2, PP3). *ARID1A*-variant was not reported in GnomAD or ClinVar databases.

Methods

DNA was isolated from fetal skin tissue sample using phenol-chloroform extraction. Total of 4432 of clinically significant genes were sequenced by Next Generation Sequencing including 20 bp of 5’ and 3’ intronic regions. 1 ref. libraries were generated using Sophia Genetics CCE_A_v1 and sequenced by NextSeq sequencer (Illumina) using 2x151 bp paired-end sequencing technology. Bioinformatics was done by Sophia DDM program (v5.7.0, Sophia Genetics). Reference genome was GRCh37/hg19. The limitations of the method used includes repeat sequence annotation, genomic rearrangements such as translocations, copy number variants smaller than 2 exons and deep intronic variants. Pseudogenes and regions with segmental duplications might not be reliably analyzed with this method.

Variant classification was performed according American College of Medical Genetics and Genomics (ACMG) classification guidance (1,2). Variants were written according Human Genome Variation Society (HGVS) standard guidance. No other findings of clinical significance were found by next generation sequencing analysis. The reference sequence for *SMARCB1* was NM_003073.4 and for *ARID1A* the reference sequence was NM_006015.4.

The clinical data, MRI and ultrasound pictures of the two pregnancies and the fetal autopsies were collected from the Turku University Hospital medical records. The ultrasound and autopsy pictures were combined in single pictures with the help of Photopea.

Data Availability Statement

Variant details are available publicly on ClinVar with accession numbers SCV002562255 (SMARCB1) and SCV002562257 (ARID1A).

A permit to publish these cases with pictures was signed in a written informed consent form by the parents.

References:

1. Kalia SS, Adelman K, Bale SJ et al. Recommendations for reporting of secondary findings in clinical exome and genome sequencing, 2016 update (ACMG SF v2.0): a policy statement of the American College of Medical Genetics and Genomics. Genet Med 2017 192. 2016 Nov;19(2):249–55.

2. Richards S, Aziz N, Bale S et al. Standards and Guidelines for the Interpretation of Sequence Variants: A Joint Consensus Recommendation of the American College of Medical Genetics and Genomics and the Association for Molecular Pathology. Genet Med [Internet]. 2015 May 8 [cited 2022 Aug 22];17(5):405. Available from: /pmc/articles/PMC4544753/
